# Genetic Structure of the Tree Peony (*Paeonia rockii*) and the Qinling Mountains as a Geographic Barrier Driving the Fragmentation of a Large Population

**DOI:** 10.1371/journal.pone.0034955

**Published:** 2012-04-16

**Authors:** Jun–hui Yuan, Fang–Yun Cheng, Shi–Liang Zhou

**Affiliations:** 1 Landscape Architecture College of Beijing Forestry University, Haidian District, Beijing, China; 2 National Flower Engineering Research Center, Key Laboratory for the Genetic and Breeding of Forestry Trees and Ornamental Plants, Beijing, China; 3 State Key Laboratory of Systematic and Evolutionary Botany, Institute of Botany, the Chinese Academy of Sciences, Haidian District, Beijing, China; 4 Gansu Forestry Technological College, Tianshui, Gansu, China; Brigham Young University, United States of America

## Abstract

**Background:**

Tree peonies are great ornamental plants associated with a rich ethnobotanical history in Chinese culture and have recently been used as an evolutionary model. The Qinling Mountains represent a significant geographic barrier in Asia, dividing mainland China into northern (temperate) and southern (semi–tropical) regions; however, their flora has not been well analyzed. In this study, the genetic differentiation and genetic structure of *Paeonia rockii* and the role of the Qinling Mountains as a barrier that has driven intraspecific fragmentation were evaluated using 14 microsatellite markers.

**Methodology/Principal Findings:**

Twenty wild populations were sampled from the distributional range of *P. rockii*. Significant population differentiation was suggested (*F_ST_* value of 0.302). Moderate genetic diversity at the population level (*H_S_* of 0.516) and high population diversity at the species level (*H_T_* of 0.749) were detected. Significant excess homozygosity (*F_IS_* of 0.076) and recent population bottlenecks were detected in three populations. Bayesian clusters, population genetic trees and principal coordinate analysis all classified the *P. rockii* populations into three genetic groups and one admixed Wenxian population. An isolation-by-distance model for *P. rockii* was suggested by Mantel tests (*r* = 0.6074, *P*<0.001) and supported by AMOVA (*P*<0.001), revealing a significant molecular variance among the groups (11.32%) and their populations (21.22%). These data support the five geographic boundaries surrounding the Qinling Mountains and adjacent areas that were detected with Monmonier's maximum-difference algorithm.

**Conclusions/Significance:**

Our data suggest that the current genetic structure of *P. rockii* has resulted from the fragmentation of a formerly continuously distributed *large population* following the restriction of gene flow between populations of this species by the Qinling Mountains. This study provides a fundamental genetic profile for the conservation and responsible exploitation of the extant germplasm of this species and for improving the genetic basis for breeding its cultivars.

## Introduction

The patterns of genetic structure in plants are the result of many interacting factors, including climatic fluctuations, complicated landforms, soil types and human activities; however, a more important factor is the evolutionary history of a species. The Qinling Mountains represent an important geographic barrier in Eastern Asia that divides the current mainland of China into southern and northern and temperate and semi–tropical regions. Additionally, these mountains are a major watershed of the Yellow and Yangtze Rivers. These regional divisions have a huge impact on numerous geographic, climactic and agricultural factors [1]. The Qinling Mountains extend for nearly 2,500 kilometers in the east-west direction and are located in a key tectonic position that links the Dabie Mountains in the east with the Qilian and Kunlun Mountains in the west [2]. Due to their large area, diversified topography, and varied climates and habitats, the Qinling Mountains have undoubtedly contributed to the evolutionary diversification of the Eastern Asian flora and fauna. High species richness and many endemic species, including more than 1,620 endemic Chinese plant species and a total of 3,124 plant species, are found in this area [1], [2]. Previous studies on the giant panda (*Ailuropoda melanoleuca*) revealed that limited gene flow occurs between the Qinling population and other populations of this species [3]. Furthermore, 46 bird species, representing 45% of all Chinese endemics, are found in this region. These findings indicate that the geological characteristics of these mountains have led to complementary evolutionary and ecological isolation and, thus, the high richness of the endemic species [4]. Research on endemic species and their distribution patterns is of great importance for understanding species diversity, conservation, and biodiversity [1], [5]. The divergence patterns giving rise to endemic species are complicated and are closely related to geology, climate, and the process of bio-evolution. Due to the complex landforms, physiognomy and many other environmental features of this area, most of its endemic plant species exhibit complex patterns with respect to the genetic diversity, structure and evolutionary history of their populations [6]–[8]. However, given the geographical and biological significance of the Qinling Mountains as geographic barriers, studies on plants in this area are lacking.

Tree peonies are an important group of plants that are referred to as ‘the king of flowers’ due to their rich palette of horticultural varieties and deep ethnobotanical history in Chinese culture; in addition, this group of plants has recently been used as an evolutionary model [9], [10]. These species are valuable ornamental and medicinal plants commonly found in China, although they are observed in other parts of the world. Approximately 2,000 cultivars of tree peonies are grown throughout the world, and more than 1,000 cultivars are found in China [9]–[12]. Tree peonies belong to section *Moudan* of the genus *Paeonia* in the family Paeoniaceae, and all 9 species of these plants are endemic to China [9]–[15]. These nine species, *Paeonia jishanensis*, *P. suffruticosa*, *P. cathayana*, *P. ostii*, *P. qiui*, *P. decomposita*, *P. rockii*, *P. delavayi* and *P. ludlowii*, are distributed in the Yunnan, Sichuan, Gansu, Shaanxi, Shanxi, Henan, Hubei, Anhui and Xizang (Tibet) provinces [9]–[15]. *Paeonia rockii* is endemic to the Qinling Mountains and the adjacent area; additionally, this species is associated with the widest distribution range and the greatest numbers of extant plants among all nine species [9]–[11]. Additionally, *P. rockii* is one of the most important ancestral species that has contributed to establishing the cultivated tree peonies. It includes two allopatric infraspecific taxa, *P. rockii* ssp. *rockii* (subspecies *rockii*) and *P. rockii* ssp. *atava* (subspecies *atava*), based on their morphological characteristics [12]–[15]. In China, subspecies *rockii* is found in the western and eastern parts of the Qinling Mountains and has been observed in the Bashan Mountains in Hubei province, whereas subspecies *atava* is limited to only the northern slopes of the Qinling Mountains and further northward in this vicinity [9]–[12]. These subspecies can be easily identified based on morphological differences: subspecies *atava* presents ovate (or rounded) and totally (or mostly) lobed leaflets, whereas subspecies *rockii* exhibits lanceolate to ovate–lanceolate and totally (or mostly) unlobed leaflets [9]–[12], [15].

Molecular phylogeographic approaches examining both nuclear and organellar genomes have become popular for understanding the patterns of population genetic structure [16], [17]. A recent report showed that the four groups (the western, eastern and northern groups and the Taibai Mountain group) of *P. rockii* closely coincide with the geographic distribution of this species, as revealed by three chloroplast genes, suggesting that the high Qinling Mountains have acted as a geographic barrier contributing to this phylogeographic structure [10]. However, the results also showed a few disparities that the clade of the Taibai Mountain (TM) population of subspecies *atava*, which is located on a northern slope of the Qinling Mountains, is more closely related genetically to the clades of the populations of the western group of subspecies *rockii* than to the clades of other populations (i.e., the Heshui and Tongchuan populations) of the northern group of subspecies *atava*. In addition, the clade of the Ganquan population (GQ) of subspecies *atava* that is located to the north of the Qinling Mountains is much closer to the clades of the Luanchuan population (LC) of the eastern group, which is located in the Xun'er Mountains of the eastern Qinling Mountains [10]. As we known, the chloroplast genome shows only a single gene genealogy and is easily affected by various kinds of demographic events (e.g., bottlenecks, vicariance, and the accidental loss of lineages) [18], [19]. In addition, the occurrence of lineage sorting and ancestral polymorphism has been proposed in *Paeonia* [10], [20]. Comparison to chloroplast markers, the nuclear microsatellite markers with relatively more fast evolutionary rates are more useful for providing a higher resolution with respect to phylogeographic events [21], [22] and are highly informative regarding genetic drift and determining bottleneck effects [23]–[25]. Moreover, several microsatellite markers with moderate to high levels of genetic variation within and among tree species have been detected [9], [26], and using these more informative markers will reveal the nature of the population structure of *P. rockii* in greater detail.

The distribution of *P. rockii* has recently been decreasing, similar to that of all other tree peony species, and it has been listed as an endangered species in the Chinese Red Data Book [27]. The major reasons for the reduced distribution in this species are habitat destruction and genetic fragmentation [10], [12]. Habitat fragmentation is becoming important to molecular ecologists and conservation geneticists, as it alters genetic diversity, which ultimately changes the entire population structure. Evidence regarding the role of genetic factors in population extinction is increasing [28]–. Given the important role of *P. rockii* in breeding new cultivated tree peonies and because it is currently facing endangered status, continuing study of the genetic diversity and genetic structure of this species is worthwhile. The original development of landforms of the Qinling Mountains was promoted by Yanshanian tectonic events in the latest Cretaceous approximately 70 Ma (million years ago) and the subsequent lateral extrusion of the Tibet plateau in the early Cenozoic (approximately 40 Ma). Additionally, its relatively stable landforms (with a gentle hillside and flat lands) formed in the early Quaternary period (2.4–1.2 Ma). Then, the present high topographic gradients with a height drop of 1,000–2,000 m in the Qinling Mountains and the highest peak, Taibai Mountain, which is 3767 m, resulted from the latest rapid uplift activated by the Himalaya orogenic belt during the Pleistocene (approximately 0.7 Ma) [1], [2]. Studies of *P. rockii* in this region could provide a good model for examining the intraspecific evolutionary processes and dynamics associated with this history of complicated landform development in the Qinling Mountains. Furthermore, these studies could provide fundamental genetic information for developing effective and sustainable conservation plans and material for the breeders of this important ornamental plant. Therefore, the major objectives of the present study were 1) to reveal the genetic diversity and population genetic structure of *P. rockii* using nuclear markers (microsatellite markers) and compare the similarities and differences between the results obtained from chloroplast DNA (cpDNA) and nuclear microsatellite markers and 2) to determine whether and how the Qinling Mountains as a geographic barrier has deeply impacted the population evolution of the endemic species *P. rockii*.

## Materials and Methods

### Sampling, DNA extraction and microsatellite amplification and scoring

**Figure 1:**
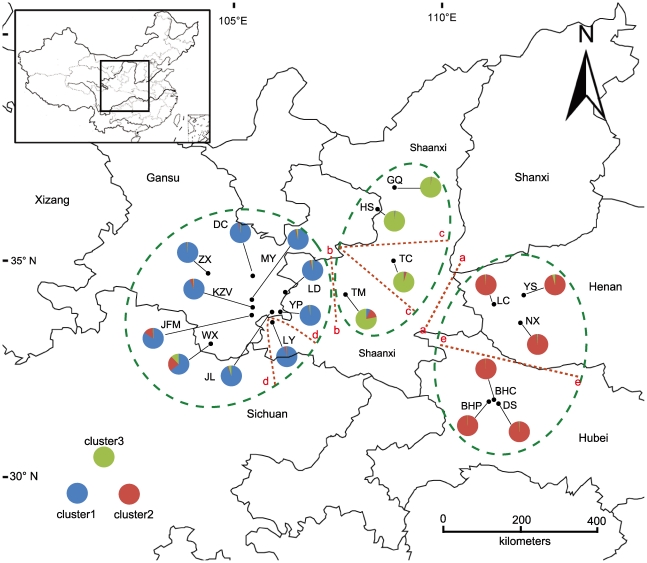
Geographic and topographic barriers, ancestral gene pools, and locations of populations sampled in *Paeonia rockii*.

*Paeonia rockii* is a deciduous shrub that grows up to 1.8 m tall, flowers from late April to May, and fruits from late July to August [10]–[11], [15]. Additionally, it is an obligate outcrossing species [31]. Samples were collected during flowering plants from April 2007 to May 2009 across the entire distribution range for this species: the entire Qinling Mountains, including from the western, northern and eastern slopes; Hubei; Shaanxi; and the southeastern part of the Gansu province. To avoid sampling closely related plants and to obtain the greatest possible genetic representation, a minimum distance of 20 m was always maintained between individuals. Because the populations of this species are still declining due to human overexploitation, only 3–30 individuals were collected from each population [11]. Fresh leaves were collected from individual adult plants of *P. rockii* (including both subspecies *rockii* and subspecies *atava*) and immediately placed in silica bags. The samplings included 335 wild individuals of 20 populations that contained four populations (GQ, HS, TC and TM) of subspecies *atava* and the other 16 populations of subspecies *rockii*, as shown in Figure 1 (Table S1. All necessary permits were obtained for the described fields.)

For each sampling site, the altitude, longitude and latitude were recorded using a GPS (eTrex Venture, Garmin Ltd., Olathe USA) (Table S1). The genomic DNA was extracted from 100 mg of dried leaf tissue using a modified cetyltrimethylammonium bromide (CTAB) method [32]. The polymerase chain reaction (PCR) analysis of the *P. rockii* collection was carried out using 14 microsatellite markers, as shown in Table S2 [9]. The 14 microsatellite markers were selected from 59 microsatellite loci developed for *Paeonia* [9], [26]. To ensure the integrity and specificity of the PCR, the products were sequenced using an ABI 3730xl automated sequencer (Applied Biosystems, Foster City, CA, USA).

PCR amplifications were performed in 10-µL reaction volumes containing 1× PCR buffer (10 mM Tris–HCl [pH 8.3], 50 mM KCl, and 1.5 mM MgSO_4_), 2.5 mM MgCl_2_, 0.2 mM each dNTP, 0.25 µM reverse primer and 0.25 µM forward primer tagged with JOE, NED, FAM or PET fluorescent dye to facilitate post-PCR multiplexing. The PCR amplifications were performed using an initial denaturation at 94°C for 3 minutes, followed by 25 cycles of 30 seconds of denaturation at 94°C, 30 seconds of annealing at 54°C and 1 minute of polymerization at 72°C, with a final extension step of 10 minutes at 72°C. The PCR products were detected using an ABI 3730xl DNA Analyzer with GeneScanTM-600 LIZ (Applied Biosystems, Foster, California, USA) as an internal size standard, and each locus was genotyped with GeneMapper ver. 4.0 (both supplied by Applied Biosystems, Foster, USA). Samples in which the alleles were uncertain were double-checked by repeating the amplification and proofreading the raw data.

### Data analysis

For each microsatellite locus, the genetic diversity was analyzed to calculate the total number of alleles (*TA*), observed heterozygosity (*H_o_*), average genetic diversity (*H_S_*) both within populations and among populations and total gene diversity (*H_T_*) [33]. For each population, the genetic diversity was measured across all of the loci in terms of the observed number of alleles (*A_O_*), observed heterozygosity (*H_O_*), unbiased expected heterozygosity (*H_E_*), private alleles (*AP*) and allelic richness (*AR*) [34]. The allelic richness (*AR*_[10]_) was standardized for 5 individuals (by discarding all of the populations with <5 individuals) using FSTAT version 2.9.3.2 [35].

The significance of any deviations from Hardy-Weinberg equilibrium, as indicated by deviations of the fixation index (*F_IS_*) [36], was tested by randomization using FSTAT version 2.9.3.2 [35]. Genotypic disequilibrium was tested for all of the loci in each population by randomization, and the obtained *P*-values ( = 0.05) were adjusted using a sequential Bonferroni correction [37] to avoid false positives with FSTAT version 2.9.3.2 [35].

To examine the geographic gradients in the genetic diversity within populations, the relationships between the genetic diversity within populations (*AR* and *H_E_*) and their geographic locations (latitude and longitude) were tested by linear regression. Reduction in the effective population size was estimated using Wilcoxon's signed-rank tests (one-tailed) with the null hypothesis, *H_E_*>*H_EQ_*, and the alternative hypothesis, *H_E_*<*H_EQ_*, under the assumption of mutation-drift equilibrium in the infinite-alleles model (IAM) and the stepwise mutation model (SMM) using BOTTLENECK version 1.2.02 [38]–[40].

The genetic differentiation among populations was estimated at each locus and across all of the loci by calculating *F_ST_* using GenAlEx version 6.2 [41]. The presence of isolation-by-distance (IBD) patterns in population differentiation was investigated by a Mantel test [42] with 1000 random permutations to evaluate the correlation between the matrix of pairwise genetic distances measured by *F_ST_*/(1−*F_ST_*) according to Rousset [43], [44]. To investigate the geographic structure of genetic variation further, a hierarchical analysis of molecular variance (AMOVA) was performed using ARLEQUIN version 3.10 [45], while the total genetic variance was partitioned into covariance components at different levels, including among groups, among populations and within populations.

The pattern of genetic variation among populations was assessed using three different methods. First, the software package, STRUCTURE 2.1 [46], applied a Bayesian method to infer the number of clusters (*K*) without using prior information on the individual sampling locations. This program distributes the individuals among *K* clusters based on their allelic frequencies and estimates the posterior probability of the data given each particular *K*. STRUCTURE was run for the *K* = 1 to *K* = 30 clusters. Each run was pursued for 10^6^ MCMC iterations, with an initial burn-in of 5×10^5^, and an ancestry model that allowed for admixture, with the same alpha for all of the populations. To assess stability, 10 independent simulations were performed for each *K*. The final posterior probability of *K*, ln*P*(*x|K*), was computed, as suggested by Pritchard et al. [46], using the analyses with the highest probability for each *K*. ln*P*(*x|K*) usually plateaus or increases slightly after the ‘right’ *K* is reached. Thus, following Evanno et al. [47], Δ*K* was calculated. The sensitivity of the final result to the specific previous assumptions of alpha and with regard to the independence of the allelic frequencies was also computed. The second method was a distance-based clustering analysis using a neighbor-joining tree based on genetic distance (*D_A_*) [33] using POWERMARKER version 3.25 [48]; the third method was a principal coordination analysis (PCA) based on allele frequencies using the Multivariate Statistical Package (MVSP) version 3.13b (Kovach Computing Services, Anglesey, Wales, UK).

The genetic barriers associated with each geographical location and population were investigated using Monmonier's maximum-difference algorithm [49] in BARRIER version 2.2 [50]. MICROSATELLITE ANALYSER (MSA) version 4.05 [51] was used to determine the genetic boundaries, and their significance was estimated using bootstrap values based on 2,000 permuted distance matrices.

## Results

### Overall genetic diversity and diversity within populations of *P. rockii*

Estimates of the diversity among the microsatellite loci are shown in Table 1. The total number of alleles per locus in the entire germplasm ranged from 5 to 22, with a total of 183 alleles. The average genetic diversity (*H_S_*) among the loci was 0.516 and ranged from 0.245 to 0.648. Similarly, the average overall genetic diversity (*H_T_*) estimate among the populations was 0.749, ranging from 0.288 to 0.899. The mean number of alleles per locus was 15.9 and ranged from 1.71 (TC) to 24.71 (JL). The mean values for the observed heterozygosity (*H_O_*) and unbiased expected heterozygosity (*H_E_*) were 0.459 and 0.492, respectively. The value of the private alleles per population (*AP*) was 2.3, and the mean within the population allelic richness (*AR*_[10]_) was 2.65, as shown in Table 2. As shown in Table 1, the *F_IS_* over all the loci was found to be significant in eight populations (BHC, DC, DS, HS, JL, KZV, LD, and ZX), whereas the *F_IS_* within populations was significant for eight loci (pdel05, pdel22, pdel33, pdel35, jx02-2, jx05-2, jx17, and jx29), as shown in Table 2. These results suggest a significant deviation from the Hardy-Weinberg equilibrium. However, no evidence of significant genotypic linkage disequilibrium was observed in any of the 1,820 permutation tests among 92 locus pairs in each population. Therefore, the 14 loci analyzed were determined to be sufficiently independent for use in Bayesian clustering.

**Table 1:** Genetic diversity measures estimated using 14 microsatellite loci in each of 20 *Paeonia rockii* populations.

Population	n	*A*	*AR*[10]	*H_O_*	*H_E_*	*F_IS_* a	IAM^b^	SMM^b^
BHC	6	1.93	1.81	0.25	0.319	0.236	0.064	0.18
BHP	6	3.29	2.71	0.469	0.391	−0.227	0.055	0.151
DC	25	5.29	2.86	0.493	0.554	0.116	0.38	0.999
DS	22	4.29	2.6	0.38	0.494	0.235	0.38	0.979
GQ	29	3.71	2.64	0.543	0.519	−0.046	0.002**	0.768
HS	26	3.21	2.13	0.347	0.395	0.122	0.428	0.979
JFM	4	3.43	-	0.673	0.691	0.03	-	-
JL	27	6.64	3.58	0.543	0.667	0.189	0.010*	0.979
KZV	13	4.64	3.16	0.509	0.609	0.17	0.052	0.866
LC	12	2.71	2.43	0.507	0.434	−0.178	0.001**	0.055
LD	24	4.21	2.6	0.476	0.533	0.11	0.034	0.709
LY	3	2.07	-	0.333	0.41	0.222	-	-
MY	24	5.36	3.12	0.616	0.642	0.041	0.001***	0.749
NX	24	4.07	2.48	0.552	0.512	−0.068	0.148	0.955
TC	2	1.43	-	0.286	0.274	−0.067	-	-
TM	3	1.86	-	0.381	0.352	−0.103	-	-
WX	30	5.14	2.77	0.538	0.566	0.051	0.047*	0.905
YP	26	4.79	2.62	0.508	0.515	0.014	0.665	1
YS	3	2.29	-	0.393	0.488	0.241	-	-
ZX	26	3.43	2.29	0.384	0.471	0.188	0.086	0.787
Average	16.75	3.69	2.65	0.459	0.492			

Note: n, sample size; *A*, number of alleles per locus; *AR*_[10]_, mean within-population allelic richness for a standardized sample size of ten gene copies; *H_O_*, observed heterozygosity; *H_E_*, unbiased expected heterozygosity; *F_IS_*, fixation index. Sequential Bonferroni correction was used to determine significance levels in multiple tests.

a Deviations of *F_IS_* from 0 in each population were evaluated by permutation tests.

b Probabilities associated with Wilcoxon's signed-rank tests (one-tailed) for *H_E_*>*H_EQ_*, where *H_EQ_* is the heterozygosity expected at mutation-drift equilibrium under the infinite-allele model (IAM) and the stepwise-mutation model (SMM) (Cornuet and Luikart 1996; Piry et al. 1999).

* *P*<0.05,

** *P*<0.01,

*** *P*<0.001.

**Table 2:** Genetic diversity measures estimated at each of 14 microsatellite loci across the 20 *Paeonia rockii* populations.

Marker	*TA*		*H_O_*	*H_S_*	*H_T_*	*F_IS_* a	*F_ST_* a	*R_ST_* a
pdel02-2	5	16.250	0.301	0.245	0.288	−0.288	0.153	0.16
pdel05	11	15.700	0.425	0.533	0.705	0.203	0.245	0.312
pdel06	8	16.500	0.798	0.566	0.79	−0.412	0.218	0.032
pdel07	12	15.600	0.688	0.648	0.756	−0.044	0.095	-
Pdel20	18	16.100	0.533	0.53	0.743	−0.077	0.3	0.495
pdel22	9	16.200	0.343	0.479	0.731	0.221	0.308	0.224
pdel29b	9	16.700	0.582	0.5	0.74	−0.165	0.334	0.467
pdel33	17	15.800	0.414	0.609	0.805	0.428	0.263	-
pdel35	13	16.200	0.49	0.554	0.792	0.137	0.33	0.207
jx02-2	22	16.300	0.505	0.627	0.899	0.203	0.337	0.399
jx05-2	13	15.050	0.235	0.324	0.715	0.229	0.584***	0.179
jx17	18	15.700	0.417	0.612	0.91	0.293	0.262	0.379
jx27	15	14.750	0.564	0.629	0.891	0.03	0.265	0.33
jx29	13	15.700	0.304	0.371	0.723	0.298	0.494***	0.64
Mean	9.15	16.250	0.471	0.516	0.749	0.08	0.302	0.487

Note: *TA*, total number of alleles; *H_O_*, observed heterozygosity; *H_S_*, gene diversity; *H_T_*, overall gene diversity; *F_IS_*, fixation index; *F_St_* and *R_ST_*, measures of relative genetic differentiation among populations under the infinite-allele model and the stepwise-mutation model, respectively. Sequential Bonferroni correction was used to determine significance levels in the multiple tests.

a Deviations of *F_IS_*, *F_ST_* and *R_ST_* from 0 were evaluated by permutation tests.

*** *P*<0.001.

No significant relationships existed between the genetic diversity in terms of the *AR* within populations (or *H_E_*) and their geographic locations (latitude and longitude). According to Wilcoxon's signed-rank test performed using BOTTLENECK, three populations (DC, GQ and LC) showed a significant deviation (excess heterozygosity) from the mutation-drift equilibrium under the IAM (after sequential Bonferroni correction), as shown in Table 3, suggesting that decreases in the population size had occurred due to a bottleneck effect.

**Table 3:** Results of the analysis of molecular variance (AMOVA) for 20 populations of *Paeonia rockii* categorized into three geographical regions.

Source of variation	d.f.	Sum of squares	Variation components	Variation (%)
Among groups	2	358.542	0.77859	16.04***
Among populations within group	17	565.003	1.00544	20.72***
Within populations	315	1891.071	3.06892	63.24***

d.f., degrees of freedom.

*** *P*<0.001, *P*-values were obtained after 11024 permutations.

The genetic diversity was found to be relatively high, but no significant differences between subspecies *atava* (*AR*_[12]_ = 2.38; *H_O_* = 0.442; *H_S_* = 0.456) and subspecies *rockii* (*AR*_[12]_ = 2.69; *H_O_* = 0.493; *H_S_* = 0.523) were observed after 1000 permutation tests (*P*>0.05).

### Genetic differentiation

As shown in Table 1, the population differentiation was significant at each locus, with an average *F_ST_* value of 0.302 detected, although the *F_ST_* ranged from 0.095 (pdel07) to 0.584 (jx05-2). The measure of the relative genetic differentiation for the pairwise *F_ST_* values between populations was found to be significant (*P*<0.05, Table S3), except for three pairwise populations (JFM-JL, JFM-MY, and BHC-BHP). The highest value of *F_ST_* of 0.57 was found between TC and BHC, and the lowest *F_ST_* value of 0.01 was found between JFM and JL. However, no significant difference between ssp. *atava* (*F_ST_* = 0.368) and ssp. *rockii* (*F_ST_* = 0.284) was detected at fourteen microsatellite loci (1000 permutations, *P*>0.05).

The Mantel test revealed a significant and strong correlation between the geographic and genetic distances of the populations (*R^2^* = 0.369, *P*<0.001; 1). The AMOVA results for the microsatellites were also significant (*P*<0.001, Table 3), revealing that 67.45% of the total variance was partitioned within populations, whereas 11.32% and 21.22% of the total variance were explained by differences among the groups (WG, NG, EG) and among the populations within groups, respectively.

### Population structure of *P. rockii*

**Figure 2:**
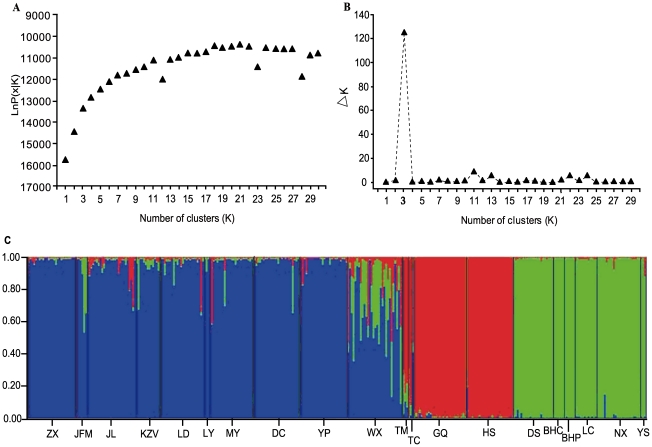
Bayesian inference of the number of clusters (*K*) of *Paeonia rockii*. *K* was estimated using (A) the posterior probability of the data given each *K* (10 replicates) and (B) the distribution of *▵K*, and (C) the three colored clusters were detected from STRUCTURE analysis. The name of each site is given below.

When using the Bayesian-based clustering approach of Pitchard et al. [46], the inference of the number of gene pools (*K*) was not straightforward because log-likelihood values for the data conditional on *K*, and ln*P*(*x|K*), increased progressively as *K* increased, as shown in Figure 2. In such a case, it may not be possible to determine the true value of *K*. However, the *▵K* values [47] computed for all *K* classes indicated a strong signal at *K* = 3, as shown in Figure 2. Changing the assumptions of an ‘equal alpha for each population’ and ‘correlated allele frequencies’ did not change this final result. The proportion of individuals in each population assigned into three clusters (or gene pools) is shown in Figure 2, and the corresponding 20 populations were plotted onto the map, as shown in Figure 1. The Bayesian clustering approach revealed that gene pool 1 (P1) was most abundant in the Western Qinling Mountains and that this pool included nine populations: JL, YP, LY, LD, KZV, JFM, DC, ZX and MY. Gene pool 2 (P2) was predominant across the Eastern Qinling Mountains (the Funiu Mountains and Xun'er Mountains in Western Henan province) and in the Shennongjia forestry area in Western Hubei province and included six populations: NX, LC, YS, DS, BHC and BHP. Gene pool 3 (P3) was predominant on the northern slope of the Qinling Mountains and in the Ziwuling forestry area and encompassed four populations: HS, GQ, TC and TM. However, only one population, WX, which was located in the southernmost region of Gansu, showed a mixture of the three ancestral genetic resources (Figure 1). With the exception of WX, it was obvious that the three gene pools were almost in accordance with the natural pattern of distribution of *P. rockii*.

The results of the distance-based clustering analysis using a neighbor-joining tree based on the *D_A_* are presented in Figure S2 and showed that the 20 populations were clustered into three major clades, except for the single clade that included WX. The western clade (WC) comprised JL, YP, LY, LD, KZV, JFM, DC, ZX and MY, whereas the eastern clade (EC) comprised NX, LC, YS, DS, BHC and BHP. Similarly, the northern clade (NC) comprised HS, GQ, TC and TM. The clustering results are in agreement with the clustering results obtained using STRUCTURE. The NJ tree also clearly showed that these three clades were distinct from each other; this result was supported by moderate to high bootstrap values of 60% for WC, 91% for EC, and 71% for NC (Figure S2).

The PCA clustered these populations into three groups, as shown in Figure S3, which corresponded to the three gene pools (Figure 2) or three clades (Figure S2). However, there was some ambiguity regarding the placement of the individuals of population WX, as the individuals of this population represented a mixture of the other groups. The three clustering results from the three different analysis methods were clearly in total agreement with each other.

### Geographic barrier among populations

**Figure 3:**
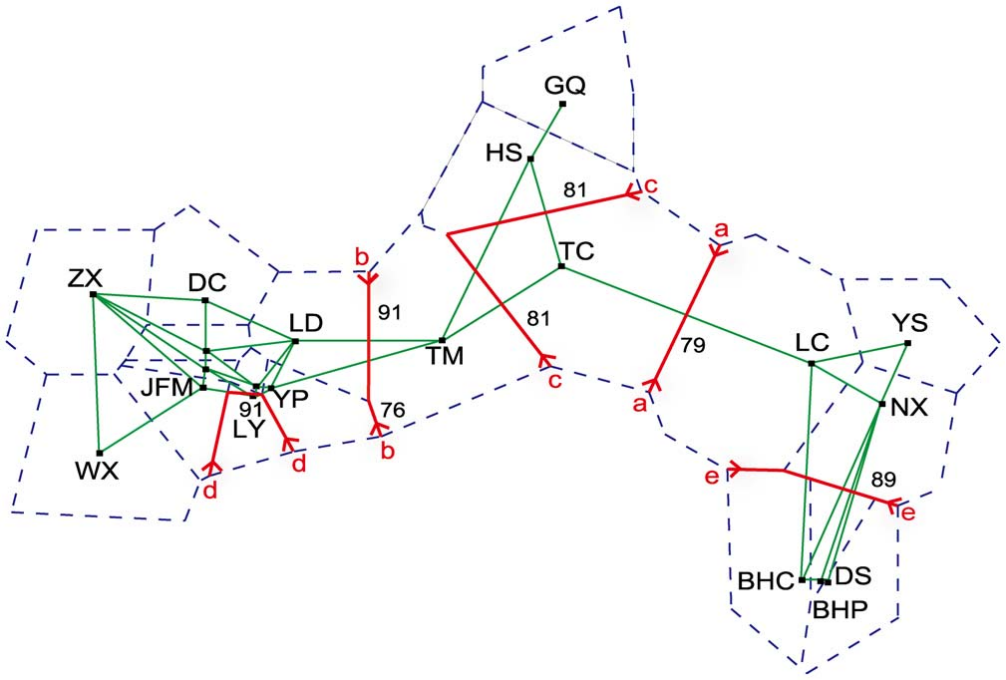
The first five genetic boundaries (thick lines) detected by BARRIER version 2.2 using genetic distance. The order of the alphabetical pairs represents the sequence of the boundary formation. Large numbers correspond to the population numbers plotted along the ordination. Small numbers represent the bootstrap values indicating support for the respective boundaries. Thin dashed lines and solid lines represent the Voronoi tessellation and the Delaunay triangulation, respectively.

Analysis of the genetic boundaries between the populations supported the five boundaries shown in Figure 1 and Figure 3. The first boundary found, between population LC and TC, separated the populations of P2 from the populations of P3 (e.g., TC) with 79% bootstrap support. The second boundary was found between TM and LD and the isolated TM population of P3 from the other populations of P1 (e.g., LD) with bootstrap values ranging from 91 to 76. The third boundary separated the populations of P1 into three groups, consisting of populations TM, TC and GQ-HS, with 81% bootstrap support. The fourth boundary isolated only one population, LY, from the other populations of P1 with 90% bootstrap support. The fifth boundary separated the populations of P2 into two groups, DS-BHC-BHP and NX-LC-YS, with 89% bootstrap support.

## Discussion

### High population differentiation, bottlenecks and the distinct genetic structure of *P. rockii*

Significant population differentiation was indicated by the high *F_ST_* values (averaging 0.302); additionally, we detected moderate genetic diversity at the population level (*H_S_* = 0.516, *H_E_* = 0.492) and high population diversity (*H_T_* = 0.749) at the species level. The finding of high genetic diversity at the species level was similar to the results obtained from three cpDNA sequences in a previous study [10] and from other molecular studies [52]–[54]. It was also suggested that the existence of restricted gene could have contributed to the development of distinct subpopulations. Bottlenecks occurred in three populations: DC, which was located in Western Qinling; LC, in the Xun'er Mountains in the eastern-to-central part of the Qinling Mountains; and GQ, in the Ziwuling forestry area far north of the Qinling Mountains. Our results indicated that population bottlenecks had occurred over the entire distributional range of *P. rockii*. The cold climatic fluctuation during the glacial cycles of the Quaternary Period might have been responsible for the whole population bottlenecks, leading to genetic drift and further increases in population differentiation. This prediction was also supported by the cpDNA data reported by Yuan et al. [10]. The subpopulations of *P. roc*kii would have subsequently diverged rapidly due to founder events, habitat fragmentation, or human disturbance. The fact that clear signals of recent bottlenecks were found in only three of the twenty populations might be attributed to the biparental inheritance characteristics of the microsatellite loci that prevented the detection of additional bottleneck events because of homoplasy characteristics or to most populations reaching a new mutation-drift equilibrium at a new effective population size following the last bottleneck events.

A significant deviation from the Hardy-Weinberg (HW) equilibrium was also observed in the *P. rockii* populations, as indicated by both the *F_IS_* value over all the loci (Table 1) and the *F_IS_* within individual populations per locus (Table 2). The main causes of the significant positive values of the *F_IS_* are the existence of excess homozygosity and inbreeding populations [55], [56]. Because *P. rockii* is an allogamous species [31], [57], the observed excess homozygosity may not be due to inbreeding; rather, it may be due to the population substructure, selection favoring homozygotes, and null alleles.

In the present study, the same clear population structure for *P. rockii*, comprising three major genetic distinct groups, P1 (the western clade), P2 (the eastern clade) and P3 (the northern clade), and only one mixed population, was detected by Bayesian analysis, Neighbor-joining tree and PCA on the basis of microsatellite data. Additionally, the genetic relationships among the populations of *P. rockii* reflecting the natural geographic locations of the populations were supported by an isolation-by-distance model for *P. rockii* using the Mantel test [40]. We found that the geographically close populations were characterized by low genetic differentiation, but as the distance among the populations increased, the genetic differentiation among the populations also increased. The observed patterns of population differentiation were also supported by the results of AMOVA (*P*<0.001), which revealed significant molecular variance both among groups (11.32%) and within groups (21.22%) (Table 3). Only population WX exhibited admixed ancestral genetic information on the basis of the microsatellite genotype data, and this population may represent the remnants of ancestral polymorphism or the original population of *P. rockii*. In addition, the population WX was found to be associated with a long branch in a cpDNA phylogenetic tree [10]. This population was located in the farthest southwestern area of the distribution range of *P. rockii*, near the range of *P. decomposita*; thus, it was worth investigating the wild germplasm at the boundary between the two species in detail to determine the evolutionary history of these populations.

### Five geographic barriers indicating that the current pattern of genetic structure is a result of the fragmentation of a panmictic *large population* of *P. rockii*

Five boundaries, consisting of two boundaries between three groups of populations (P1, P2 and P3) and three additional boundaries within the three groups, were detected using BARRIER software [50] with Monmonier's maximum-difference algorithm [49]. The detected genetic structure potentially identifies a historical limitation of migration and gene flow among and within the three groups of *P. rockii* populations. The first boundary, between the northern clade and the eastern clade, was located between the TC population of the Weihe Basin and population LC of the Xun'er Mountains in the eastern-to-central Qinling Mountains. This boundary was obvious in nature and was supported by the existence of the tectonic boundary of the Luonan-Luanchuan Fault [2]. This Fault isolated the populations of *P. rockii* in the Eastern Qinling Mountains and the Bashan Mountains from the other populations in the Central Qinling Mountains. The second boundary, between the northern clade and the western clade, was located between the TM population in the Taibai Mountains at the northern boundary of the Qinling Mountains and the LD population in the mountain area of Southern Gansu in the western part of the Qinling Mountains. As a result of the normal fault growth and footwall flexure of the mountains, especially after a large-magnitude rapid uplift in 0.7 Ma, the Taibai Mountain became the highest peak of the Qinling Mountains [1], [2]. The third boundary, corresponding to the Weihe River, was detected between the populations of the northern clade and separated the TC population of the Weihe Basin from the populations of the Taibai Mountains and Ziwuling forestry area of the Loess Plateau. The Weihe Basin, located between Taibai Mountains and the Loess Plateau, is a downfaulted basin that was produced from an upper wall extensional normal fault during the Cenozoic [2]. The fourth boundary was found within the western clade and isolated only one population, LY, on the southern slope of the Qinling Mountains from the populations of the mountainous area in Gansu. The fifth boundary was detected within the eastern clade and separated the NX-LC-YS populations of the Xun'er Mountains in the Western Qinling Mountains from the DS-BHC-BHP populations of the Bashan Mountains.

It was highly unexpected that the genetic subpopulations of *P. rockii* would be so *perfectly* in line with the existing geographical barriers. The current landform and topographical features of the Qinling Mountains mainly formed by beginning with a small-magnitude uplift of 70–40 Ma and a rapid large-magnitude uplift beginning at 1.2–2.4 Ma (or 0.7 Ma), especially with respect to the higher mountain features of the Taibai Mountains [1], [2], [5], [58]. In the last 5000 years, the climate of the Qinling Mountains has repeatedly changed, according to historical records [1], [5], [59]. The major trend of climatic change in this region has been a shortening of warming periods and a lengthening of cold periods, such that the climate type on the northern slope of the Qinling Mountains has gradually changed from subtropical to temperate [5], [59]. Following the Qing Dynasty (AD 1636–1911), the human population growth in mountainous areas, the use of new types of crops and the implementation of unreasonable development policies led to serious damage to the forestry cover and the overall deterioration of the Qinling Mountain ecosystem [5], [59]. The estimated divergence time for the *P. rockii* populations using cpDNA sequences ranges from 0.4 to 1.6 Ma according to Yuan et al. [10] and is concurrent with the time scale of the latest rapid uplifts of the Qinling Mountains. Thus, it is strongly suggested that the genetic differentiation of *P. rockii* populations is greatly related to the rapid, large-magnitude uplift of the Qinling Mountains. Although the estimation of divergence rates or times using the molecular clock hypothesis has been based on uncertain assumptions and approximate values with respect to the lack of *Paeonia* fossils, these estimations are still useful for attempts to understand the rate of evolution and the historical biogeography of *Paeonia*. In addition, geographic isolation has been proposed by Sang et al. [60] as a major driving force of speciation and rapid diversity for *Paeonia* species, and the timing of the intercontinental disjunction of peonies had been roughly estimated to 16.6 Ma in the Miocene based on cpDNA sequences [60]. We could not rule out the possibility that the genetic clusters of the *P. rockii* populations that were detected based on both microsatellite and cpDNA data were merely random coincidence; however, it is clear that the geographic isolation that has occurred in the Qinling Mountains has contributed to limiting the gene flow between the *P. rockii* populations [9], [10].

How and why has this type of genetic structure developed in *P. rockii*? We hypothesize that a formerly continuously distributed panmictic *large population* of *P. rockii* has fragmented into small, isolated populations. Such changes would lead to an erosion of the genetic variation and an increase of genetic differentiation among populations due to enhanced genetic drift and reduced gene flow [29], [30]. This hypothesis was supported by a series of findings. An isolation-by-distance model for *P. rockii*, based on the Mantel test, indicated a significant correlation between the genetic distance and the geographic distance. The recent bottleneck events detected, the high degree of population differentiation among three gene pools and the existence of five natural geographic barriers suggest that the genetic differentiation among the studied populations has been impacted by impediments to gene flow and that geographic distance has played an important role in the current genetic pattern (Figure 1 and Figure 3).

The other possible causes leading to the three distinct *P. rockii* gene pools are inefficient pollen flow, low seed dispersal, and low germination rates across the distributional range of this species [10], [52], [54], [61]. These results suggest that the geographically close populations of *P. rockii* exhibited a low genetic differentiation, but as the distance among the populations increased, the genetic differentiation among the populations also increased. These differences with respect to the genetic differentiation are due to the geographic barriers that have separated the different gene pools, which each subsequently evolved individually.

Although many lines of evidence presented above support the hypothesis that the population structure in *P. rockii* can be mainly explained by a simple isolation-by-distance model associated with geographic barriers, this hypothesis requires the evaluation of additional factors that may have influenced the observed genetic population structure, such as natural selection. Environmental factors also contribute to the current population differentiation to some degree, and it is worth continuing to investigate how these populations that are isolated by natural geographic barriers adapt to their niches and evolve independently.

### Comparison of molecular and morphological variation in *P. rockii*

When the genetic groups, as determined based on the microsatellite loci, were compared with the groups based on cpDNA sequences, the results were essentially in agreement [10]. However, there were two points of disparity: the first was that the TM population was grouped into the northern clade using the SSR (simple sequence repeats) data, but it was indicated as an independent clade, isolated from the other populations of the northern group and closer to the western clade in the cpDNA tree [10]; the second point was that the GQ population was grouped into the northern clade using the SSR data but into the eastern clade in the cpDNA tree [10]. In general, different evolutionary histories and events are reflected in different molecular markers, such that the SSRs, which are representative of the nuclear genome, mainly reflect pollen flow, and the cpDNA sequences, which are representative of the chloroplast genome, only reflect seed flow in *Paeonia* [9], [21]–[23]. The SSR data are suitable for reflecting the relatively recent intraspecies evolutionary historical events, ranging from at least 3,000–30,000 to more than 200,000 years [62]–[65], whereas the cpDNA sequences are more suitable for detecting long-term events, such as more than 16.6 Ma in *Paeonia* and tracing the evolutionary history for all higher plant species across millions of years [60], [66]. However, the chloroplast genome shows only a single gene genealogy [18] and is easily affected by various types of demographic events (e.g., bottlenecks, vicariance, and the accidental loss of lineages) [17], [19]. In addition, lineage sorting and ancestral polymorphism has been proposed to occur in *Paeonia* [10], [20] and was also suggested by the results of this study; furthermore, lineage sorting and/or ancestral polymorphism were responsible for the first disparity related to population TM [9]–[12], [20], as mentioned above. The second point of disparity, associated with population GQ, reflects one evolutionary event of long-distance seed dispersal reported by Yuan et al. [10].

Phenotypic variation followed the geographical distribution of this species. The populations that are geographically distributed in the north of Qinling Mountains and further north in its vicinity (populations TM, GQ, HS and TC) exhibit ovate or rounded and totally or mostly lobed leaflets, classified as *P. rockii* ssp. *atava*. However, the other populations of this species, mainly distributed in the eastern or western part of the Qinling Mountains, present lanceolate or ovate–lanceolate and totally or mostly unlobed leaflets, classified as *P. rockii* ssp. *rockii*. With respect to phenotypic variation, the nuclear genes, reflected in SSR data, and environmental factors are both responsible for the morphological development of *P. rockii*; however, the chloroplast genome is not. Thus, the results of this study support the idea that a more comprehensive understanding of evolutionary history and speciation is provided by the integrated analysis of data from two genomes (nuclear and chloroplast genome).

Cultivated tree peonies, respected as ‘the king of flowers’ in China, are very popular ornamental plants, and breeding programs will greatly benefit from this study. More new cold-resistant, wet-resistant, or hot-resistant varieties with more ornamental value could be bred in the future using the different genetic germplasms from the different gene pools of *P. rockii* because the isolated gene pools of this species might have accumulated a number of mutations to adapt to the different ecologic niches within the different climates after independently evolving for almost 0.7–2.4 Ma.

In conclusion, significant population differentiation and clear genetic structure were revealed in *P. rockii* in this study, suggesting the occurrence of significant subpopulation and population bottlenecks among the populations of this species. More interestingly, five geographic barriers were identified between and within three genetic groups geographically located in the Western, Eastern and Northern Qinling Mountains. It was also proposed in this report that the current genetic structure of *P. rockii* could be the result of the fragmentation of a formerly continuously distributed panmictic *large population*. Furthermore, our results provide a fundamental genetic profile for the conservation and responsible exploitation of the extant germplasm of this species to improve the genetic basis for breeding its cultivars. Finally, this study offers new insight into the abundant biodiversity of plant species that are endemic to the Qinling Mountains of China and provides a model case for studying their complex evolutionary history.

## Supporting Information

Figure S1 **Mantel test for matrix correlation between the genetic distance and log geographical distance.**
(TIF)

Figure S2 **Clustering analysis of the 20** ***Paeonia rockii*** **populations based on genetic distance.** The phylogenetic tree was constructed with the POWERMARKER software package (Liu et al., 2005) based on the populations' pair-wise genetic distances (Nei 1983). The shapes near site names on the tree indicate STRUCTURE grouping.
(TIF)

Figure S3 **Principal coordinate analysis (PCA) of** ***Paeonia rockii*** **populations based on microsatellite data.**
(TIF)

Table S1 **Population locations and sample sizes for** ***Paeonia rockii*** **.**
(DOC)

Table S2 **Repeat motifs, annealing temperatures (** ***Ta*** **), primer sequences and the range of alleles detected per locus for the microsatellite loci.**
(DOC)

Table S3 **A pair-wise matrix of geographic distance (upper right) and** ***F_ST_*** **(below left)_between the 20 populations of** ***Paeonia rockii*** **.**
(DOC)
