# Supplementary figures and images for: Genetic Structure of the Tree Peony (Paeonia rockii) and the Qinling Mountains as a Geographic Barrier Driving the Fragmentation of a Large Population
Source: PLoS One. 2012 Apr 16;7(4):e34955. doi: 10.1371/journal.pone.0034955 (PMC3327690; doi:10.1371/journal.pone.0034955)

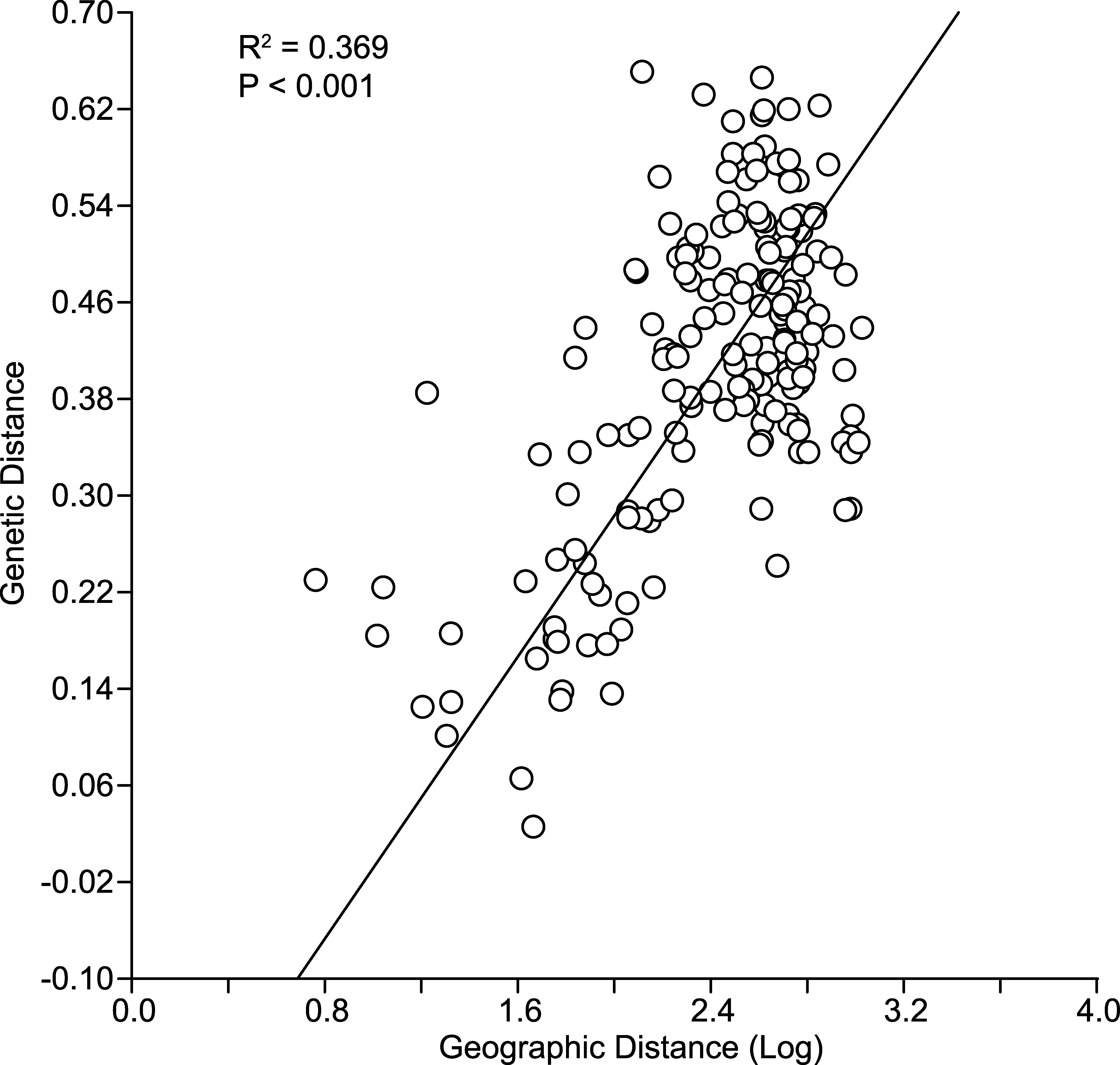

Supplement: Figure S1 — Mantel test for matrix correlation between the genetic distance and log geographical distance. (TIF) [file pone.0034955.s001.tif]

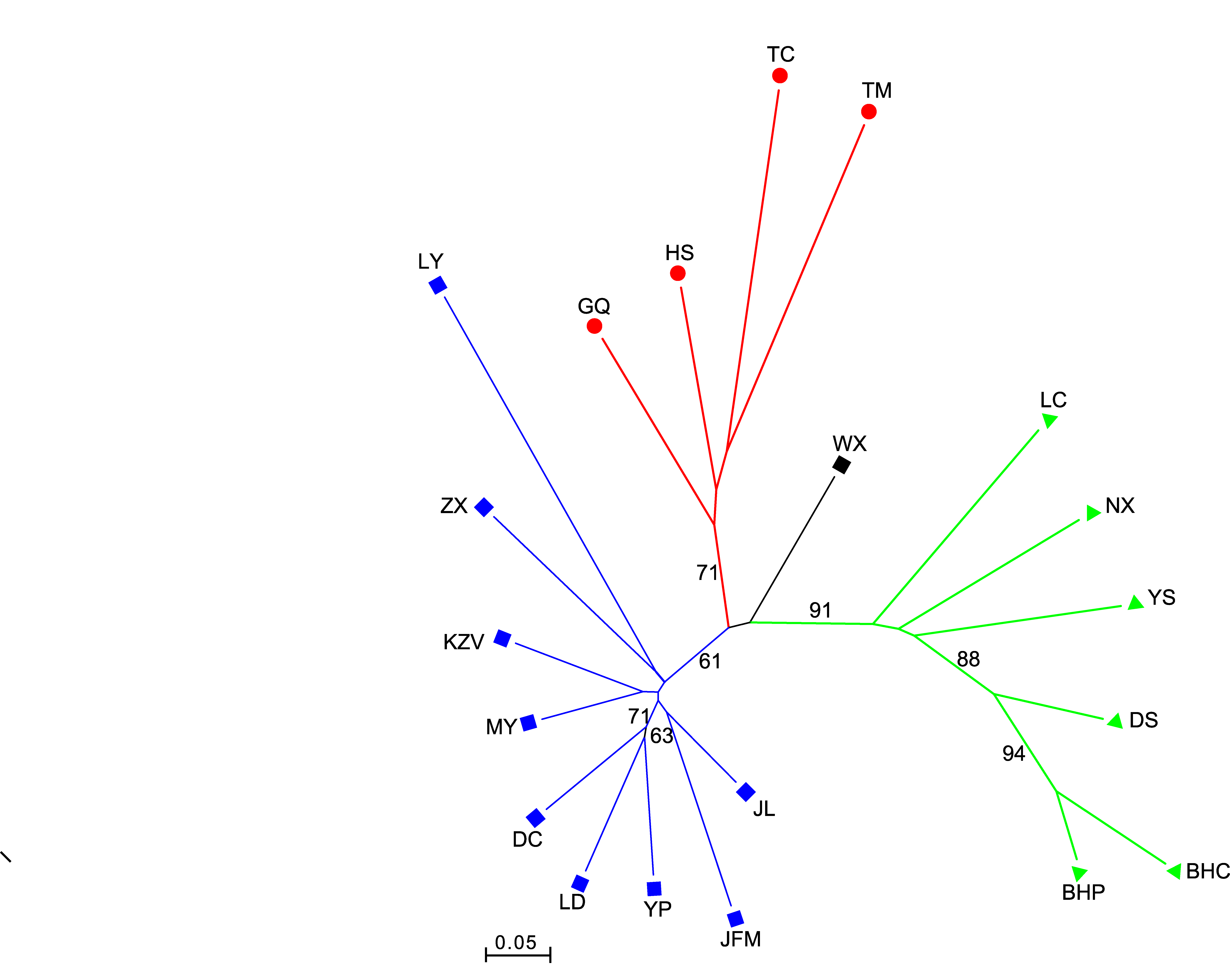

Supplement: Figure S2 — Clustering analysis of the 20 Paeonia rockii populations based on genetic distance. The phylogenetic tree was constructed with the POWERMARKER software package (Liu et al., 2005) based on the populations' pair-wise genetic distances (Nei 1983). The shapes near site names on the tree indicate STRUCTURE grouping. (TIF) [file pone.0034955.s002.tif]

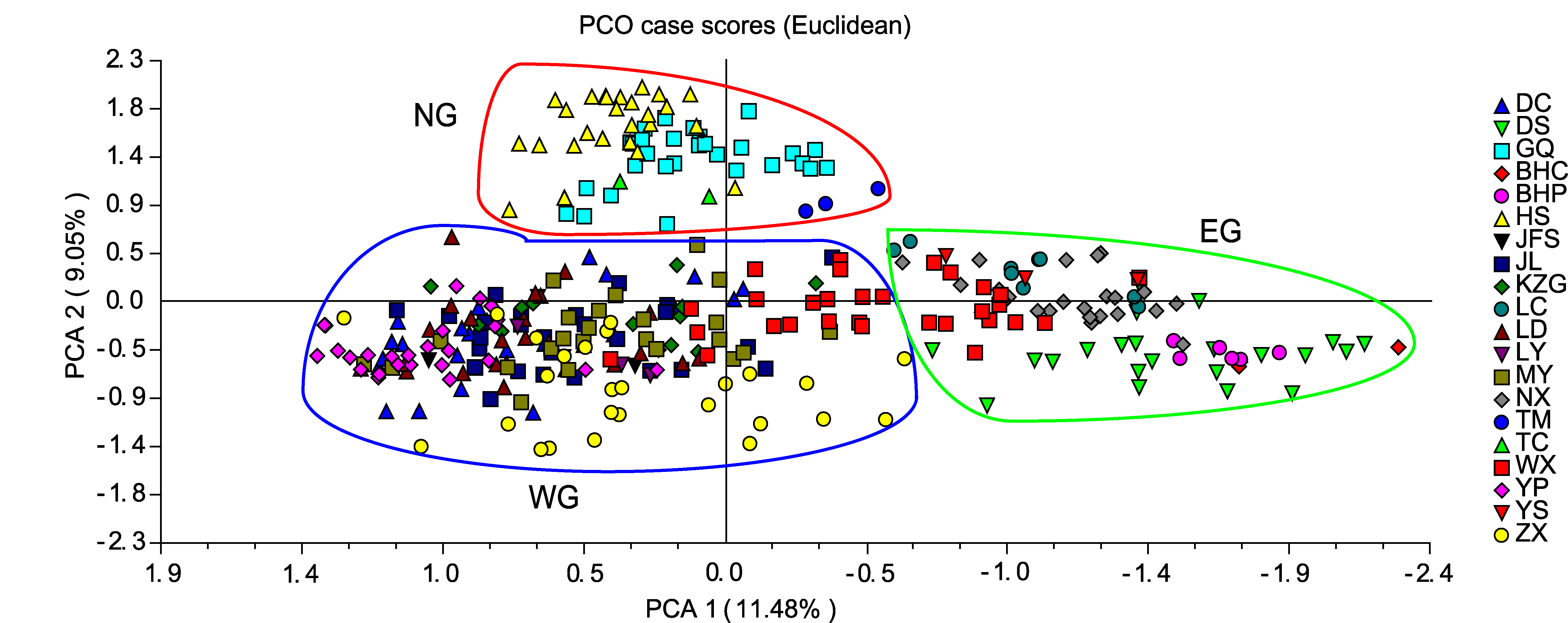

Supplement: Figure S3 — Principal coordinate analysis (PCA) of Paeonia rockii populations based on microsatellite data. (TIF) [file pone.0034955.s003.tif]
