# Supplementary material for: Genetic Structure of the Tree Peony (Paeonia rockii) and the Qinling Mountains as a Geographic Barrier Driving the Fragmentation of a Large Population
Source: PLoS One. 2012 Apr 16;7(4):e34955. doi: 10.1371/journal.pone.0034955 (PMC3327690; doi:10.1371/journal.pone.0034955)
Supplement: Table S1 — Population locations and sample sizes for Paeonia rockii . (DOC) [file pone.0034955.s004.doc]

## Supporting information

**Table S1.** Population locations and sample sizes for *Paeonia rockii*.

| **Population** | **N** | **Locality** | **LAT**  **(N)** | **LON**  **(E)** | **Elevation** |
| --- | --- | --- | --- | --- | --- |
|  |  |  |  |  | **(m)** |
| BHC | 6 | Henchong Mountain, Baokang county, Hubei province | 31.72 | 111.12 | 1742 |
| BHP | 6 | Changchongya, Baokang county, Hubei provice | 31.72 | 111.29 | 1377 |
| DC | 25 | Dangchuan town, Tianshui city, Gansu province | 34.57 | 105.7 | 1473 |
| DS | 22 | Dashui Forestry farm, Baokang county, Hubei province | 31.69 | 111.35 | 1599 |
| JFM | 4 | Jifeng Mountain, Chengxian county, Gansu province | 33.69 | 105.67 | 1739 |
| JL | 27 | Jialing town, Huixian county, Gansu province | 33.69 | 106.17 | 1286 |
| KZV | 13 | Kongzigou Valley, Chengxian county, Gansu province | 33.87 | 105.7 | 1359 |
| LC | 12 | Xuner Mountain, Luanchuan county, Henan province | 33.93 | 111.21 | 1150 |
| LD | 24 | Zhangjia town, Liangdang county, Gansu province | 34.15 | 106.52 | 1587 |
| LY | 3 | Lueyang county, Shaaxi province | 33.59 | 106.13 | 1332 |
| MY | 24 | Mayan town, Huixian county, Gansu province | 34.05 | 105.7 | 1401 |
| NX | 24 | Baotianman, Neixiang county, Henan province | 33.51 | 111.85 | 1458 |
| WX | 30 | Liujiaping town,Wenxian county, Gansu province | 33.01 | 104.74 | 1675 |
| YP | 26 | Yanping village, Huixian county, Gansu province | 33.67 | 106.29 | 1575 |
| YS | 3 | Yang Mountain, Songxian county, Henan province | 31.87 | 115.65 | 1200 |
| ZX | 26 | Zhangxian county, Gansu province | 34.63 | 104.67 | 1911 |
| GQ | 29 | Xiashi town, Guanquan county, Shaanxi province | 36.58 | 108.95 | 1402 |
| HS | 26 | Taibai town, Heshui county, Gansu province | 36 | 108.65 | 1337 |
| TC | 2 | Tongchuan City, Shaanxi province | 34.91 | 108.93 | 1128 |
| TM | 3 | Taibai Mountain, Baoji city, Shaanxi Province | 34.16 | 107.84 | 1400 |
| Average | 16.75 |  | - | - | - |
| Total | 335 |  | - | - | - |

Note: Sampling site (code and location), sample size (N), latitude (LAT), longitude (LON), elevation (in meters above sea level), mean number of alleles per locus (Allele no.) and polymorphism indel content (PIC) for each population from the 20 collection sites are presented. *﹡,* populations that have experienced a bottleneck, as indicated by a significant excess of genetic diversity in relation to allelic diversity under the infinite allele model (IAM) (*P* < 0.05)
