# Supplementary material for: Genetic Structure of the Tree Peony (Paeonia rockii) and the Qinling Mountains as a Geographic Barrier Driving the Fragmentation of a Large Population
Source: PLoS One. 2012 Apr 16;7(4):e34955. doi: 10.1371/journal.pone.0034955 (PMC3327690; doi:10.1371/journal.pone.0034955)
Supplement: Table S2 — Repeat motifs, annealing temperatures ( Ta ), primer sequences and the range of alleles detected per locus for the microsatellite loci. (DOC) [file pone.0034955.s005.doc]

Supporting information

**Table S2**. Repeat motifs, annealing temperatures (*Ta*), primer sequences and the range of alleles detected per locus for the microsatellite loci.

| Locus | Repeat motif | Size (bp) | *Ta* (°C) | Primer sequences (5’-3’) |
| --- | --- | --- | --- | --- |
| Pdel02-2 | (AC)5 | *229-246* | *54* | F: ATGCTACAGCCACTACACTACAC |
|  |  |  |  | R: TCTTCTCCTCCCTTTCTCCTCT |
| Pdel05 | (AG)15 | *201-229* | *50* | F: CCAATGTGGAAAATGAGTT |
|  |  |  |  | R: CAAGCACAAGATGTAAGAA |
| Pdel06 | (AG)19 | *214-232* | *54* | F: TGGATTCTTATTTGTTTTGAG |
|  |  |  |  | R: ACACCGTGTAGCAGATGATGA |
| Pdel07 | (AG)30 | *247-287* | *48* | F: TATCATTCTAACGGTGGTT |
|  |  |  |  | R: GAGGTAGATACTGGAACTT |
| Pdel20 | (TC)6 | *276-340* | *54* | F: TATAAATGGGAAGCAGACTCAA |
|  |  |  |  | R: TATACTCAGCCTCGAAAAGAAG |
| Pdel22 | (AG)9 | *276-320* | *54* | F: TCGCCCAACCTGTCGTGGAGAT |
|  |  |  |  | R: TTGAATAGAGCGGAATGGAAAA |
| Pdel29b | (TGG)6 | *224-260* | *54* | F: CTGCCATTTCTTGCCTTCTTTGT |
|  |  |  |  | R: TCTACCCTGCCAACAGCACATAC |
| Pdel33 | (TC)10 | *131-165* | *54* | F: ACGGACTAAATTGCAGTGAGC |
|  |  |  |  | R: AGAACAAATCAGACGGCGAGA |
| Pdel35 | (GA)10 | *286-314* | *54* | F: ATGTCACCGAAAGTTGTGC |
|  |  |  |  | R: AAAGCCTGGTGCAGTTATT |
| Jx02-2 | (TC)9 | *302-354* | *54* | F: TTGGTTGGTGAAGGTGTT |
|  |  |  |  | R: CTTCGATAACCGCAGGAGGAT |
| Jx05-2 | (CT)17 | *232-260* | *54* | F: GCCACAAGAAAACAAAAACC |
|  |  |  |  | R: CCTTCACCACTACTTCCCCAT |
| Jx17 | (TC)20 | *218-270* | *54* | F: CAAACTACCTGAATGTTCGGCTC |
|  |  |  |  | R: CATCAAATTACCAAAGAAATCCT |
| Jx27 | (TC)5 | *303-323* | *48* | F: GTTATAGAACCACTGACAT |
|  |  |  |  | R: TGAGAGACAAATAATCGTG |
| Jx29 | (TC)17 | *392-454* | *54* | F: TTTCCTTATTTGAGTTTGGGTGAG |
|  |  |  |  | R: ATCATGTTTTCGAGAGAAGCATC |

rockii

|  |  |  |  |  |  |  |  |  |  |  |  |  |  |  |  |  |  |  |  |  |
| --- | --- | --- | --- | --- | --- | --- | --- | --- | --- | --- | --- | --- | --- | --- | --- | --- | --- | --- | --- | --- |
|  |  |  |  |  |  |  |  |  |  |  |  |  |  |  |  |  |  |  |  |  |
|  |  |  |  |  |  |  |  |  |  |  |  |  |  |  |  |  |  |  |  |  |
|  |  |  |  |  |  |  |  |  |  |  |  |  |  |  |  |  |  |  |  |  |
|  |  |  |  |  |  |  |  |  |  |  |  |  |  |  |  |  |  |  |  |  |
|  |  |  |  |  |  |  |  |  |  |  |  |  |  |  |  |  |  |  |  |  |
|  |  |  |  |  |  |  |  |  |  |  |  |  |  |  |  |  |  |  |  |  |
|  |  |  |  |  |  |  |  |  |  |  |  |  |  |  |  |  |  |  |  |  |
|  |  |  |  |  |  |  |  |  |  |  |  |  |  |  |  |  |  |  |  |  |
|  |  |  |  |  |  |  |  |  |  |  |  |  |  |  |  |  |  |  |  |  |
|  |  |  |  |  |  |  |  |  |  |  |  |  |  |  |  |  |  |  |  |  |
|  |  |  |  |  |  |  |  |  |  |  |  |  |  |  |  |  |  |  |  |  |
|  |  |  |  |  |  |  |  |  |  |  |  |  |  |  |  |  |  |  |  |  |
|  |  |  |  |  |  |  |  |  |  |  |  |  |  |  |  |  |  |  |  |  |
|  |  |  |  |  |  |  |  |  |  |  |  |  |  |  |  |  |  |  |  |  |
|  |  |  |  |  |  |  |  |  |  |  |  |  |  |  |  |  |  |  |  |  |
|  |  |  |  |  |  |  |  |  |  |  |  |  |  |  |  |  |  |  |  |  |
|  |  |  |  |  |  |  |  |  |  |  |  |  |  |  |  |  |  |  |  |  |
|  |  |  |  |  |  |  |  |  |  |  |  |  |  |  |  |  |  |  |  |  |
|  |  |  |  |  |  |  |  |  |  |  |  |  |  |  |  |  |  |  |  |  |
|  |  |  |  |  |  |  |  |  |  |  |  |  |  |  |  |  |  |  |  |  |
