# Supplementary material for: Genetic Structure of the Tree Peony (Paeonia rockii) and the Qinling Mountains as a Geographic Barrier Driving the Fragmentation of a Large Population
Source: PLoS One. 2012 Apr 16;7(4):e34955. doi: 10.1371/journal.pone.0034955 (PMC3327690; doi:10.1371/journal.pone.0034955)
Supplement: Table S3 — A pair-wise matrix of geographic distance (upper right) and FST (below left)_between the 20 populations of Paeonia rockii . (DOC) [file pone.0034955.s006.doc]

## Supporting information

***Table S3.*** *A pair-wise matrix of geographic distance (upper right) and F_ST_ (below left)_between the 20 populations of P.* rockii

|  | BHC | BHP | DC | DS | GQ | HS | JFS | JL | KZG | LC | LD | LY | MY | NX | TC | TM | WX | YP | YS | ZX |
| --- | --- | --- | --- | --- | --- | --- | --- | --- | --- | --- | --- | --- | --- | --- | --- | --- | --- | --- | --- | --- |
| BHC |  | 16.07 | 595.21 | 21.06 | 575.53 | 528.19 | 554.42 | 511.82 | 559.6 | 246.73 | 508.08 | 512.11 | 568.44 | 211.31 | 409.49 | 408.62 | 615.74 | 501.63 | 428.06 | 681.66 |
| BHP | 0.07 |  | 608.65 | 5.78 | 581.13 | 535.14 | 569.05 | 526.23 | 573.99 | 246.76 | 521.55 | 526.66 | 582.58 | 206.71 | 417.55 | 420.61 | 631.25 | 516.01 | 412.02 | 695.59 |
| DC | 0.40* | 0.34* |  | 614.43 | 368.89 | 311.68 | 97.8 | 106.89 | 77.73 | 509.83 | 87.14 | 114.48 | 56.66 | 577.95 | 297.42 | 201.47 | 193.66 | 113.32 | 971.71 | 94.5 |
| DS | 0.14* | 0.16* | 0.31* |  | 585.95 | 540.23 | 574.73 | 531.94 | 579.71 | 250.29 | 527.33 | 532.35 | 588.32 | 208.82 | 422.84 | 426.36 | 636.68 | 521.72 | 407.52 | 701.35 |
| GQ | 0.43* | 0.41* | 0.30* | 0.36* |  | 68.78 | 437.99 | 408.46 | 421.42 | 357.29 | 348.02 | 419.22 | 406.36 | 430.22 | 184.42 | 287.02 | 551.17 | 404.15 | 807.43 | 442.54 |
| HS | 0.52* | 0.49* | 0.31* | 0.43* | 0.30* |  | 374.46 | 342.83 | 358.72 | 326.73 | 282.99 | 353.45 | 344.85 | 402.22 | 123.71 | 218.31 | 488.23 | 338.03 | 791.98 | 391.52 |
| JFM | 0.38* | 0.33* | 0.09* | 0.27* | 0.28* | 0.31* |  | 46.22 | 20.19 | 511.16 | 93.4 | 42.81 | 41.2 | 572.09 | 328.89 | 206.75 | 113.97 | 56.44 | 953.48 | 139.96 |
| JL | 0.32* | 0.28* | 0.12* | 0.26* | 0.24* | 0.29* | 0.01 |  | 47.79 | 465.09 | 60.9 | 11.02 | 59.74 | 525.87 | 287.79 | 162.57 | 152.15 | 10.41 | 907.94 | 173.64 |
| KZG | 0.36* | 0.30* | 0.11* | 0.27* | 0.26* | 0.28* | 0.05* | 0.09* |  | 507.16 | 81.25 | 49.05 | 21.11 | 569.73 | 318.24 | 199.69 | 129.75 | 58 | 954.26 | 127.53 |
| LC | 0.37* | 0.34* | 0.32* | 0.28* | 0.34* | 0.41* | 0.32* | 0.26 * | 0.31* |  | 432.39 | 470.64 | 506.71 | 76.05 | 234.78 | 310.25 | 607.19 | 455.16 | 474.46 | 604.32 |
| LD | 0.39* | 0.35* | 0.14* | 0.30* | 0.27* | 0.35* | 0.11* | 0.09 * | 0.14* | 0.30* |  | 71.86 | 75.33 | 497.82 | 237.02 | 122.27 | 207.05 | 58.42 | 888.31 | 176.9 |
| LY | 0.51* | 0.46* | 0.28* | 0.40* | 0.40* | 0.47* | 0.21* | 0.19* | 0.25* | 0.45* | 0.28 |  | 64.14 | 530.5 | 296.77 | 170.41 | 143.42 | 16.72 | 910.78 | 176.45 |
| MY | 0.33* | 0.29* | 0.11* | 0.25* | 0.26* | 0.27* | 0.03 | 0.08* | 0.07* | 0.30* | 0.16* | 0.23* |  | 570.93 | 310.81 | 197.16 | 145.7 | 68.85 | 958.41 | 114.36 |
| NX | 0.36* | 0.29* | 0.31* | 0.26* | 0.37* | 0.40* | 0.30* | 0.27* | 0.28* | 0.29* | 0.33* | 0.44* | 0.29* |  | 310.06 | 376.78 | 662.66 | 515.68 | 399.75 | 672.17 |
| TC | 0.57* | 0.53* | 0.37* | 0.44* | 0.33* | 0.38* | 0.31* | 0.29* | 0.31* | 0.48* | 0.34* | 0.45* | 0.31* | 0.46* |  | 130.69 | 439.94 | 280.07 | 709.23 | 389.96 |
| TM | 0.52* | 0.49* | 0.38* | 0.39* | 0.34* | 0.41* | 0.37* | 0.31* | 0.38* | 0.43* | 0.37* | 0.43* | 0.35* | 0.43* | 0.44* |  | 313.59 | 153.79 | 770.53 | 295.44 |
| WX | 0.33* | 0.31* | 0.23* | 0.24* | 0.27* | 0.34* | 0.20* | 0.19* | 0.18* | 0.28* | 0.27* | 0.35* | 0.15* | 0.30* | 0.39* | 0.40* |  | 160.13 | 1030.38 | 180.08 |
| YP | 0.45* | 0.41* | 0.13* | 0.35* | 0.32* | 0.35* | 0.12* | 0.12* | 0.15* | 0.35* | 0.11* | 0.31* | 0.16* | 0.37* | 0.40* | 0.43* | 0.29* |  | 897.54 | 183.09 |
| YS | 0.32* | 0.28* | 0.27* | 0.21* | 0.29* | 0.39* | 0.23* | 0.22 | 0.24* | 0.15* | 0.27* | 0.40* | 0.25* | 0.20* | 0.46* | 0.43* | 0.24* | 0.32* |  | 1064.86 |
| ZX | 0.43* | 0.41* | 0.25* | 0.35* | 0.35* | 0.43* | 0.20* | 0.21 | 0.25* | 0.38* | 0.28* | 0.35* | 0.18* | 0.40* | 0.47* | 0.46* | 0.25* | 0.30* | 0.36* |  |

**P* < 0.05
